# The Contribution of Applied Social Sciences to Obesity Stigma-Related Public Health Approaches

**DOI:** 10.1155/2014/267286

**Published:** 2014-03-24

**Authors:** Andrea E. Bombak

**Affiliations:** Department of Community Health Sciences, University of Manitoba, S113-750 Bannatyne Avenue, Winnipeg, MB, Canada R3E 0W3

## Abstract

Obesity is viewed as a major public health concern, and obesity stigma is pervasive. Such marginalization renders obese persons a “special population.” Weight bias arises in part due to popular sources' attribution of obesity causation to individual lifestyle factors. This may not accurately reflect the experiences of obese individuals or their perspectives on health and quality of life. A powerful role may exist for applied social scientists, such as anthropologists or sociologists, in exploring the lived and embodied experiences of this largely discredited population. This novel research may aid in public health intervention planning. Through these studies, applied social scientists could help develop a nonstigmatizing, salutogenic approach to public health that accurately reflects the health priorities of all individuals. Such an approach would call upon applied social science's strengths in investigating the mundane, problematizing the “taken for granted” and developing emic (insiders') understandings of marginalized populations.

## 1. Introduction

Obesity stigma and negative stereotypes of obese people are widespread and damaging to the health, dignity, human rights, and quality of life of obese individuals [1]. Standard media and biomedical depictions of obese individuals contribute to this stigmatization by positing that obesity incidence is nearly entirely dependent on individualistic actions [1]. Furthermore, obese individuals' may occupy numerous intersecting social roles and identities based on their gender, class, race, and other social positions. Such intersectional processes may compound the degree of marginalization obese persons' experience.

Biomedical and media depictions invariably refer to obesity as a crisis or epidemic. Obesity's multifactorial and multilevel etiology is reduced to an “energy balance” model of causation, which inadequately explains weight trajectories [2]. Despite the oft-reported inefficacy of weight loss dieting, public health almost invariably recommends weight loss [3, 4].

Interventions may be unsuccessful due to a narrow focus on weight loss, an overly simplistic notion of how obese individuals live; experience their bodies; the contexts they inhabit; and the opportunities available to them in seeking wellness, happiness, and a full life. Furthermore, while health is posited as the ultimate goal, these projects frequently focus on weight and deem fatness or higher weights as necessarily pathological. This is especially important given that the populations often targeted by such campaigns may differ culturally and socioeconomically from dominant groups. These diverse factors may affect their lifeways, priorities, and health conceptualizations in manners which may require in-depth exploration to produce truly beneficial and sensitive programming. Qualitative social scientists, such as anthropologists or sociologists, trained in methods such as ethnography, may be uniquely suited to explore the lived experiences of obese individuals. They may aid in developing a public health strategy that is suited to the priorities and lifestyles of all individuals and is implemented in a manner consistent with a salutogenic, positive, and holistic understanding of health promotion. This paper discusses the potential for in-depth, qualitative social science research to concretely contribute to program delivery. Even within the expanding fields of critical obesity research, as Warin and Gunson note [5], the compiling of actual obese people's experiences and perspectives has been limited. Through actual collaboration between disciplines, rather than public health researchers and programmers and qualitative social scientists operating in separate silos, programming may be developed that addresses the needs of multiple marginalized populations. Importantly, rather than reliance on stereotypes, such an approach would facilitate compassionate and evidence-based policy and programming.

## 2. Context

### 2.1. Challenges for Obese Persons in the Healthcare System

Understanding how both patients and providers view health-related topics and how these actors must negotiate these views in a care-setting context is critical to planning effective and respectful public health care delivery. Biomedical research nearly invariably posits obesity as a health crisis, despite evidence that obese individuals may be metabolically healthy; overweight people live longer than “normal” weight persons, as do obese individuals in chronic disease populations and fit obese persons compared to unfit “normal weight” persons [2, 6].

Weight bias is moderate to high among healthcare professionals and trainees, including those specializing in obesity or nutrition-related practice [7–11]. A recent systematic review of physicians' views on treating adult obesity found that physicians believed it was important to treat obesity [12]. They were confident in their skills respecting obesity treatment, although obesity knowledge was actually limited. Physicians believed they were largely unsuccessful in treating obesity but attributed this to patient noncompliance and lack of motivation, which coincides with their general view of overweight and obese individuals as lazy [12]. Similar results were attained by Foster and colleagues in 2 nationally representative American surveys (*n* = 5000) [13]. Physicians felt treatment for obesity was ineffective; held negative views of obese patients' appearance and compliance; attributed obesity causation to lifestyle factors; and sought greater compensation in delivering obesity treatment [13]. Similar to other studies, physicians in New York State expressed frustration in attempting to treat obesity [14]. This frustration was based on the extent to which obesity-contributing factors were outside their control, low sense of self-efficacy in treating obesity, and a perceived lack of reimbursement.

Physician attitudes respecting childhood obesity are comparable to those for adult obesity [15]. Physicians believe treating childhood obesity is important and provide lifestyle advice and dietitian referrals. However, they also feel they are unsuccessful in treating obesity, largely as a result of noncompliance and lack of motivation of patients. Reimbursement appeared to be less of an issue regarding physicians' views of childhood obesity [15].

Studies have also presented more nuanced views of clinicians' attitudes relating to obesity treatment. For example, physician's BMI may mediate physicians' likelihood of counseling obese patients. Among primary care physicians, weight loss discussions were more likely to be initiated by physicians who believed clients had a higher BMI than themselves [16]. Normal weight physicians were also more likely to feel confident administering said advice, to feel physicians were responsible for serving as normal weight role models and to doubt patients would trust weight-related advice from overweight or obese clinicians [16].

Another study conducted in New York City involved a chart review and patient survey, and its results suggest little focus on obesity in practice [17]. It was found that physicians were relatively unlikely to enter an official diagnosis of overweight or obesity on a patient's chart, to advice weight loss or refer to a dietitian [17]. This generally contrasted with patient wishes. Patients generally wanted to lose weight and receive physician advice and referral to a dietitian. A qualitative study on German physicians' and patients' views on obesity management found that doctors were concerned with a potential overemphasis on obesity [18]. Both physicians and patients emphasized the need for multidisciplinary approaches to obesity management, the excess burden on primary care centres, and emphasized respectful trusting relationships between practitioners and patients [18]. The need for more services and professional involvement, delivered by physicians or other providers, either separate from or within a primary care setting, was also referred to by both groups [18].

A recent debate in the Canadian Family Physician journal has highlighted that practitioners may be developing a more critical view on the orthodoxy of advocating weight loss for every obese patient. Bosomworth presents a review on possible negative mortality, morbidity, and quality-of-life outcomes of weight loss [19]. It is suggested that metabolically healthy obese individuals should strive to remain weight stable, not to gain or lose weight [19]. An accompanying editorial encourages promoting self-acceptance and healthy lifestyles for obese patients, as weight loss is nearly impossible [20]. Havrankova presents the argument that weight loss as a public health goal is futile and contends that the focus should be on preventing obesity [21, 22]. Garrel posits that obesity prevention is largely outside the practitioner's purview and argues instead for obesity treatment [23, 24]. The treatment proposed, however, involves fairly modest goals. Garrel adopts the Edmonton Obesity Staging System (EOSS) for guiding obesity treatment [25]. Based on these guidelines, Garrel supports urging weight loss only in obese individuals who have comorbid conditions; physicians should work with obese individuals without comorbid conditions to prevent weight gain [23, 24]. Treatment of obese individuals with comorbid conditions would involve treating these comorbidities, setting realistic weight goals with patients, and warning them of unsafe weight loss methods. The EOSS presents a more nuanced view of obesity than mere anthropometric measures. It allows for the possibility that some obese patients may be healthy and not benefit from treatment [25].

Dietitians are also viewed as a key factor in obesity management. Physicians are more likely to refer to dietitians than to gastric bypass surgeons or to prescribe medication [12]. Dietitians agree on their primacy in obesity treatment [26]. In a sample of 514 Canadian dietitians, about 90% felt obesity contributed to ill-health and a large majority felt obese individuals should be encouraged to lose weight. However, they also emphasized the importance of health measures other than weight in obesity treatment, and the majority advised their clients against weighing themselves [26]. Indeed, many dietitians were positively disposed toward a weight-neutral, Health-at-Every-Size (HAES) approach; however, there was variation in plans to adopt more structured eating plans and abandoning weight loss as a goal. Some also argued that certain (larger) sizes exceeded healthy limits [27].

In examining clinician perspectives on obesity, what appears to be essential, therefore, is the establishment of trusting and respectful relationships between clinicians and their obese patients in designing interventions for obese persons. These relationships must be sustained in light of obesity's likely intractability and potential nonpathological nature. A greater understanding of clinicians' and obese patients' health perspectives, perceptions, and priorities over the life course is paramount for achieving these aims. Better understanding of the experience of visiting healthcare providers as a larger patient may also provide valuable insight into sensitive care delivery and health (not weight)-centric treatment approaches.

A greater awareness of the stigma obese individuals encounter in day-to-day life, as well as in the healthcare system, is of particular importance to improve health and quality of life. Examining these issues in depth is essential to rectify a social justice issue that has immense health and quality-of-life implications for a significant population. Ethnographers and other applied social science researchers may be especially adept at exploring this issue and learning from obese persons about the oppression they may endure.

### 2.2. Weight Stigma

Obese individuals report high levels of weight discrimination in their everyday lives. This discrimination occurs in both interpersonal encounters and in institutional settings such as social situations, places of employment, and health care settings [7]. This discrimination is particularly notable among heavier individuals (BMI > 35 kg/m^2^), 40% of whom reported experiencing discrimination in the American 1995-96 National Midlife Development survey. Women and younger adults were also at considerably higher relative risk of experiencing weight stigma [7].

These findings coincide well with reports of high levels internationally of stigmatizing attitudes toward obese persons [28]. The public emphasizes the presumed causal role of the individual in developing obesity, and this was the single strongest predictor of possessing a stigmatizing attitude [28]. The pervasiveness of obesity stigma in health care settings is of particular importance for obese individuals' health and public health planning [7, 8]. Awareness of these sentiments may make it especially difficult for obese individuals to find adequate medical care [1]. Social scientists could explore these issues and seek to deploy an in-depth, exploratory perspective from obese individuals' own (emic) perspective. Ultimately, such studies may contribute to improved health care and public health strategies.

### 2.3. Portrayal of Obesity

The depiction of obesity in biomedical, media, and other popular accounts contributes to the manifestation and degree of obesity stigma present among the public. Gard and Wright identified that obesity research was communicated to the public in a way that erases the inherent uncertainty and imprecision of epidemiological studies and presents obesity as necessarily a health crisis, despite nondefinitive evidence [2]. As this work is generated from within a biomedical institution, it is presumed to be unquestionably objective and lacking in any moralizing, political, or ideological thrust [2]. Such a frame allows for the dismissal of alternate views as fallacious, whether they originate from alternate epistemological scholarly arenas, lay perspectives, or embodied experience. It also disqualifies concerns of how this research may affect or be affected by antifat bias.

These portrayals may inadvertently silence the source most important to understanding the public health programming needs of obese individuals, that is, obese individuals themselves, by presenting a homogenizing and blaming view of such individuals' lifestyles and experiences. These views are likely to be reliant on assumptions, rather than data on obese persons' lived experiences. Bringing forth obese individuals' perspectives and learning from their embodied knowledge through multidisciplinary research may enhance public health programming efforts.

Media commentators' particular moral values help to establish which component of life they choose to implicate as causing obesity. Often these values are related and presumed to be unique to modern life, such as diminished quality family time or the slothful nature of the current generation of children [2]. Similar to these accounts are evolutionary explanations for obesity stressed in media [2], which may present an inaccurate understanding of the biological or archaeological evidence of humanity's evolutionary past. Regardless of the validity of such claims, these and other arguments that invoke a golden era of nonobesity and health continue to deploy a moralistic frame that denigrates modern lifestyles as decadent, slothful, and glutinous. Particularly, adult commentators often direct their ire toward children as embodying the presumed deterioration of societal values and lifestyles [2]. Child health prevention programs reliant on this framing may risk stigmatizing, disempowering, and harming children, rather than on trying to understand the children's own perspectives.

The individualistic notion of obesity etiology remains dominant in media accounts [1]. These portrayals posit that individuals are responsible for both the cause and cure for obesity. This depiction contributes to stigmatizing attitudes toward obese individuals, who come to be viewed as embodying a remediable social and health burden, borne, in part, by others. It further discounts that obese individuals may be healthy, may choose to emphasize more holistic, less weight-centric conceptualizations of health, or may have lives that preclude investment or engagement in self-care. Interestingly, since 2003, Lawrence has detected a shift in American media coverage of obesity [29]. Personal responsibility framing dominates; however, increasing focus is being placed on environmental factors that may contribute to obesity, particularly the fast food industry. Unfortunately, this emphasis has not significantly affected antifat attitudes [28, 29]. While environmental risks for obesity are acknowledged, individuals are assumed to willingly incur these risks and thus still be largely culpable for their body size and worthy of disapprobation [29]. This discrimination has spread to previously nonstigmatizing nations [30]. Rather than castigating obese individuals for their size, qualitative social science researchers may be in a position to rewrite common stereotypical assumptions of obese individuals' lives by working collaboratively to better understand the health and experiences of an often maligned group and addressing the health concerns most relevant to them.

In addition to referring to obesity as an epidemic or crisis [2, 31], an even more extreme figurative device is employing military metaphors [32]. This discourse effectively constructs obese individuals as targets in a war or even as domestic terrorists [32]. Terms like “contagious” are also used when describing obesity in epidemiological studies using methods such as social network analysis [33, 34]. This frame seems to be particularly detrimental to the potentially health-enhancing and stress-reducing benefits of social support networks for individuals. As developing obesity is extremely feared [35, 36], it may be very isolating for obese individuals to have their friendship portrayed as a risk for developing obesity [37].

### 2.4. Risks of Weight Stigma

It has been suggested that stigma may serve as a motivator for weight loss among obese individuals [1, 38]. However, the evidence provides a far bleaker picture; stigmatizing obese individuals is an ineffective tactic in reducing obesity rates. Rather than stimulating healthful behaviors, stigma is more likely to produce poor eating habits and inactivity and thus may actually augment both obesity prevalence and disordered eating [1, 39–42]. Additionally, the catastrophic rhetoric used to describe the obesity epidemic has been suggested as a potential contributor to rising rates of eating disorders [43]. Interventions designed to ameliorate childhood obesity have also been implicated in the development of eating disorders [44–49].

Given the adverse psychological outcomes produced from weight discrimination, stigma may also compromise physical and psychological health through stress-induced neuroendocrine dysregulation [1, 50]. Muennig and colleagues found that the difference between ideal weight and actual weight had a stronger effect on mentally and physically unhealthy days than BMI in American adults [50], suggesting that body dissatisfaction may have a potent impact on health, over and above objective fatness. The health effects of stigma-induced stress will likely be exacerbated by healthcare discrimination [7, 8], consequent inadequate care, and subsequent avoidance of healthcare providers [1]. Similarly, Ernsberger reviewed the evidence on obesity, socioeconomic status, and mortality [51]. Obesity may increase the risk of poverty, downward social mobility, and subsequent ill-health through prejudice and discrimination affecting education, employment, income, housing, and healthcare opportunities [51]. Thus, weight stigma may be far more health-damaging than previously thought and far more damaging than “excess weight” [51].

Importantly, weight loss may not eliminate the consequences of stigma. For example, formerly obese adolescent girls continued to suffer from the lower self-esteem characteristic of chronically obese adolescents [52]. Additionally, there is qualitative evidence that weight loss efforts and concomitant lifestyle, dynamic, and emotional changes may result in dissolutions of friendships [41]. This may be compounded by the “contagious” framing of obesity [37]. This deterioration of social support may be an unacknowledged mechanism through which weight stigma affects health, which may previously have been erroneously attributed to the weight itself.

### 2.5. Stigma Management

In such a stigmatizing context, individuals may utilize a variety of stigma management techniques [53]. These methods may include attempting to lose weight as both an act of contrition and to minimize the fatness for which obese individuals are oppressed [54–58]. Monaghan conducted ethnography on male members of a United Kingdom slimming club and explored the effects of stigma on their lives. One method of managing stigma utilized by these individuals, who varied in their acceptance of obesity as a discredited state, involved the accounts they related concerning their weight issues [56, 58]. Some accounts entailed offering excuses that mitigated individual responsibility for obesity, such as appeals to a genetic condition or environmental issues. Other individuals justified their size through appeals to their enjoyment of food or pride in their powerful size. Individuals who rejected the discredited nature of obesity emphasized natural body diversity or deemphasized the importance of weight regarding health [56, 58]. Significantly, these accounts are socially contingent. While one account may suit a particular context, a different account may be more useful in different situations [56, 58]. To cope with stigmatizing environments, some individuals may also choose to align themselves with the fat acceptance movement [57]. Being aware of the everyday effects of stigma on individuals' lives, lifestyles, and health is an essential factor in planning salutogenic, engaging, beneficial, and inclusive public health strategies. For example, understanding obese persons' decisions to avoid particularly stigmatizing physical fitness venues may allow public health planners to design an atmosphere conducive to supporting individuals of all sizes to engage in enjoyable, health-conducive movement.

### 2.6. The Need for Obese Individuals' Perspectives

The literature thus suggests that obese individuals experience substantial stigmatization and exist in an environment saturated with nonproblematized information concerning the health risks of excess weight. This information is often presented in a manner that assumes control for health resides in the individual, the possibility of health and obesity are mutually exclusive, and that achieving wellness constitutes a moral imperative. To a greater or lesser extent, individuals appear to have adopted this discourse and allowed it to influence their perspectives and lifestyles. Individuals' health perceptions may be influenced by pervasive mainstream weight discourse but also be mediated by somatic understandings of wellbeing. As evidence suggests that obese individuals may be healthy, obese individuals may not absorb popular obesity-related messaging, based on their own somatic signaling or knowledge of their lifestyles. Individuals may also have differing priorities regarding wellness that supersede weight concerns, such as an emphasis on experiencing pleasure or mitigating income-related food insecurity. Regardless of the health risks associated with obesity, a greater understanding of how obese individuals feel concerning their health and quality of life, what obese individuals regard as their priorities concerning health, and what they feel would most benefit their quality of life and wellness is necessary. These views may also alter over time as different weight trajectories are experienced, and this is essential to consider given the chronicity of obesity. In crafting public health messaging, programs, and policies, such knowledge will be essential in creating effective and ethical interventions.

Also essential is considering the potentially multifaceted effects of stigma on obese individuals' lives [59, 60]. This includes incorporating that which is “most at stake for actors in a local social world” within the social dimensions of stigma [60, p. 1525]. For example, this may involve stigma impeding life chances, financial and life opportunities, and the fulfillment of individual or familial role functions [59, 60]. This view of stigma also considers the manner through which stigma is sociosomatic and how through psychobiological, moral-somatic, and moral-emotional pathways, stigma may have direct physiological consequences [61]. These data on obese people's perspectives and health will add to the rich work conducted in critical obesity and fat studies, theoretical understandings of the body, and qualitative research done on discursive bodily and health perspectives of individuals' of all sizes, for example, [2, 32, 36–48, 56–58, 62–68].

## 3. Alternative Models to a Weight-Centric Public Health

### 3.1. Health-at-Every-Size

Recent movements have emerged that are critical of a weight-centric public health model. One such undertaking is the Health-at-Every-Size (HAES) approach. HAES advocates promote healthy living without a focus on weight loss [3]. HAES advocates are critical of weight loss dieting's very low rates of sustained weight loss and potentially negative effects on physical and mental health. Such effects can include lowered self-esteem, heightened stress, weight cycling, and bone loss [4]. Furthermore, HAES advocates are critical of the overestimation of excess weight's effects on morbidity and mortality; the discounting of the existence of healthy obese individuals; and the ethics of promoting weight loss, given its low levels of success and possible harms [3]. Instead, the HAES movement promotes the benefits of engaging in enjoyable physical activity and body acceptance. HAES practitioners advice eating nutritionally according to an intuitive eating model, based on intuitive cues of hunger and satiety [3]. Clinical trials of HAES lifestyle interventions have demonstrated improvements in psychosocial, clinical, physiological, and behavioral measures, independent of weight loss. Critically, participants did not experience negative consequences, including weight gain, and these results compared favorably with diet-focused intervention groups [3, 4]. The inclusion of social scientists into public health strategizing would facilitate the inclusion of true insiders' (i.e., obese individuals') perceptions on the advantages and disadvantages of these novel developments.

### 3.2. Critical Obesity Scholarship

Frequently aligned with HAES, or fat acceptance advocates, are critical obesity scholars [68]. These scholars, often from outside health fields, have raised critiques concerning dominant obesity discourse. Studies critiquing the biological and epidemiological underpinnings of the prevailing view of obesity as a major health concern and the product of individual behavior have been published. A variety of issues have been raised by these scholars and include the use of terms such as epidemic or crisis in referring to obesity; the moralization of a presumed health issue and resultant ethical and stigmatizing implications of interventions and messaging; and the dominant obesity discourse's effects on individuals' bodily understandings, [2, 32, 36–48, 56–58, 62–68]. Findings from these and similar studies would benefit intervention planners in better understanding the daily lives of obese persons, obese individuals' perspectives on health, and how to plan the most beneficial interventions.

## 4. Obesity and Ethnography

### 4.1. Ethnographic Exploration of Stigma

Anthropology's traditional focus on the subaltern has become more cognizant and reflective of power relations in research than in past, more colonially complicit, eras of anthropology. A focus today on stigmatized populations, such as obese persons, would draw upon emerging strengths in anthropology, including the utilization of anthropology in social critique and the development of critical ethnography [69]. While in the contemporary social climate obese individuals are often stigmatized, obese individuals and their allies have also marshaled resistance to dominant obesity messaging. Monaghan conducted ethnography on the lives of male members of a United Kingdom slimming club [56, 58]. This provided invaluable information on the experience and management of stigma in these individuals' lives. Further investigations into the experience of weight stigma for men, women, and children are necessary to grapple with a source of discrimination, which can be life and health-damaging but has attracted limited attention and censure. Indeed, ethnographers may be especially useful in this respect. Such forms of stigma may operate largely unconsciously, and participant observation may be a valuable tool in detecting this stigma, its effects, and providing empirical support for effecting change. Further ethnography on obese individuals, with a focus on health and with applied aims, would be immeasurably useful to health care providers, public health professionals, and the general public of all sizes.

Thus, collaboration between ethnographers and health researchers in planning health interventions would serve the interests of both disciplines and more importantly the needs of obese individuals. Ethnographers could study individuals' experiences in health care settings, fitness centers, or in employment and educational settings, in which weight bias has been reported. In documenting the manner in which obese individuals' live; the choices they make; their priorities in wellness and quality of life; their contributions to society and their potential experiences with others' biases; applied social scientists could put a human face on obesity and work toward producing a positive, life-affirming, and inclusive public health focused on reducing stigma and blame and helping all people achieve a high quality-of-life.

### 4.2. Applied Outcomes of Social Science Research

Applied social science can enhance obese individuals' quality of life by providing a greater understanding and problematization of “taken for granted” assumptions regarding obese individuals' health, lives, and lifestyles. This epitomizes what Rabinow [70] views as a particularly salient and novel area for anthropological exploration. The problematization of “serious speech acts” and practices, things, and classifications. Anthropological experimentation in these areas could move aspects of a culture from being viewed as natural, to contingent, and finally, from a reflexive perspective [70, p. 67]. The problematization of taken-for-granted social attitudes and processes is particularly relevant in studying weight stigma, which is largely considered unremarkable, or even acceptable, in current sociocultural climes.

Prevailing attitudes suggest that obese individuals are necessarily unhealthy, lazy, self-indulgent, and lacking in will power [1]. Internalization of such accounts may make individuals feel unhealthy, unworthy, immoral, or disempowered based on their body size or innocuous lifestyle choices. Emic understandings of obese individuals' lives, through ethnography or similar in-depth research methods, could lead to a less-stigmatizing, more healthful view of how obese individuals navigate their everyday lives. Such knowledge could inform more effective public health strategizing.

This more in-depth understanding may contribute invaluably to public health approaches in the future. A greater understanding would be available on what constitutes health to obese individuals, what they prioritize in terms of wellbeing, and their experiences in seeking to live well lives. This perspective would help counter potentially traumatic past experiences of dieting or healthcare discrimination; produce a more patient-centered approach more congruent to obese individuals' lives and wishes; and help establish trusting relationships and bonds between obese individuals, health care providers, and public health officials. All these aims are essential for any collaborative health venture to proceed. Applied social scientists, particularly those with access to health practitioner and policy-related audiences, may be especially valuable in this research endeavor with respect to disseminating findings and facilitating progress. These individuals may circulate a critique to key stakeholders in health-related fields, who are in a position to effect change [69].

Specific opportunities for qualitative researchers could involve participant observation of obese persons' interactions with the healthcare system to identify what these persons find the most challenging and most promising aspects of their care in these settings. Critical ethnography could also be conducted within existing prevention programs to identify successful, sensitive, or problematic components of existing programs. Multisited ethnography could allow obese persons to identify sites of stigmatization and places of support for undertaking self-care.

### 4.3. Challenges and Facilitators for Applying Ethnographic Research

Needless to say, challenges would arise in attempting a critical ethnography of the lived experience of obese individuals. Investment in orthodox obesity understandings are entrenched among biomedical, public health, and lay audiences. Seeking to conduct and disseminate such research may prove difficult. This may be particularly evident in trying to demonstrate its value to those largely unaware of obesity stigma's pervasiveness, its effects, or who have internalized negative stereotypes of obese persons. Researchers would also have to be reflexive concerning their own biases and truly and accurately reflect the findings of research participants. In order to best stimulate change, these researchers would also have to strive to create positive, open-minded, and collaborative relationships with a variety of biomedical, public health, and lay audiences in order to exchange findings in a manner most conducive to cooperation and reform. Otherwise, researchers risk merely circulating critique among those already critical of dominant obesity discourse or incapable of initiating reform to public health interventions and messaging [69].

Facilitating the collaboration of partnerships between patient-providers and policy makers would be of great value. This would allow policy makers to learn from patients' experiences and desires to rewrite public health messaging, programs, and clinical guidelines to better address obese persons needs and priorities in terms of healthcare system allocation; address systemic and interpersonal discrimination; and establish weight industry regulations more in keeping with respect for consumers and truth in advertising. These possibilities, along with numerous others, would allow researchers and public-health planners to learn what health priorities are pertinent to a needlessly patholigized and stigmatized population. It may open the doors to previously nonconsidered structural reforms and the provision of holistic, local health needs.

## 5. Conclusions

Exposure to obesity discourse is likely inescapable for obese individuals. These individuals are inundated daily with messages concerning the risk that they embody, solely by virtue of their size. These risks are thought to extend beyond their own health, to the health of others, the sustainability of the health care system, and even society's future. Furthermore, society deems them as culpable for their presumed poor health and for straining the health care system. Within this environment, widespread and largely acceptable weight stigma has become normalized. Exposure to such messaging renders obese individuals as a population worthy of special consideration in planning public health programs to avoid reinforcing demeaning stereotypes. What are lacking, despite this oppressive focus on obesity, are the perspectives of obese individuals themselves concerning their health, goals, wishes, and quality of life. Furthermore, limited attempts have been made to bridge communicative gaps between physicians and obese patients and to gain a more thorough view of physicians' perceptions on obesity. Given applied social scientists' dedication to understanding emic perspectives and expertise in in-depth exploratory methods such as ethnography, they may be particularly suited to aid in this pursuit. The outcome may be beneficial, inclusive, and nonstigmatizing public health programs from which all individuals would greatly benefit.
